# Unveiling the effect of CaCl_2_ on amyloid β aggregation *via* supercritical angle Raman and fluorescence spectroscopy and microscopy†

**DOI:** 10.1039/d4cp00996g

**Published:** 2024-10-01

**Authors:** Nathalia Simea Münch, Subir Das, Stefan Seeger

**Affiliations:** a Department of Chemistry, University of Zurich Winterthurerstrasse 190 8057 Zurich Switzerland sseeger@chem.uzh.ch

## Abstract

Amyloid β aggregation is an important factor in Alzheimer's disease. Since calcium homeostasis plays an important role in amyloid β aggregation, it is crucial to study the interaction between calcium ions and amyloid β directly at the surface of the lipid membrane. With supercritical angle techniques, the signal of interest at the surface is easily separated from the bulk solution, making them a powerful tool for aggregation study. In this work, the influence of calcium ions on amyloid β aggregation over different aggregation time periods is investigated with supercritical angle Raman and fluorescence spectroscopy and microscopy. Note that calcium ions have a larger influence on amyloid β_1–42_ than on the 40 amino acid variant. We found that a small layer of calcium ions significantly protects the lipid membrane against the protein insertion process.

## Abstract 2

The effect of calcium ions with amyloid beta on a solid supported bilayer is studied by supercritical angle Raman and fluorescence spectroscopy and microscopy. The results give insight into understanding protein structure and aggregation behavior.

## Graphical abstract

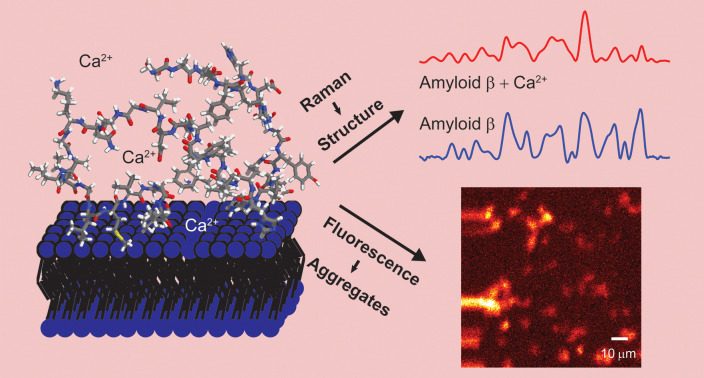

## Introduction

One of the main neurodegenerative diseases related to aging is Alzheimer's disease (AD), which affects approximately 50 million people worldwide. Two major pathological hallmarks are commonly associated with AD: (1) the aggregation of amyloid β (Aβ) to neurotoxic oligomers and fibrils with a subsequent accumulation of amyloid plaques outside the neurons.^1–3^ (2) The aggregation of tau protein that forms neurofibrillary tangles in the grey matter of the brain.^4^ Due to this protein deposition, the lipid membrane is disrupted, which leads to neural degeneration. The most important consequence is the loss of crucial brain functions such as motion, learning ability, memory or language skills.^5^ As a state-of-the-art diagnosis of AD, the detection of such Aβ aggregation is necessary for early diagnosis.^6–8^

Calcium ions are important for modulating the functions of neuron cells, *e.g.* neurotransmitter release or forming new synapses.^9^ Therefore, Ca^2+^-binding proteins ensure calcium homeostasis inside neurons by regulating the amount of Ca^2+^ ions inside the cells. Otherwise, reactive oxygen species (ROS) accumulate on the neurons, which leads to cell death.^2^ Furthermore, calcium ions strongly interact with the membrane's lipid phosphate groups. Stronger interactions occur with negatively charged phosphatidylserine (PS), such as POPS or DOPS. These interactions decrease the negative charge of the lipid membrane.^10,11^ Due to the decrease of negative charges, the electrostatic attractions between lysine and the phosphate head groups are hindered, aggravating the approach of the peptide to the membrane. However, other positive amino acids such as histidine or arginine can also form attracting forces with negative lipid head groups, blocked by calcium ions. Y. Yang *et al.*^11^ showed a combination of hydrophobic and electrostatic interactions between membrane bound peptides and lipids *via* all-atom molecular dynamics simulations. The first step is the electrostatic approach of the peptide towards the lipid bilayer. Close to the membrane, the hydrophobic residues, especially phenylalanine, are anchored into the hydrophobic part of the lipid bilayer. The calculations further show a strong affinity of Ca^2+^ to the phosphate groups enhancing the positive charge of the membrane.^11^ Similar observations were also introduced by Sciacca *et al.*^12^ using NMR spectroscopy to test the insertion of amyloid peptides into lipid membranes. In the presence of calcium ions, the fibril insertion into the membrane is harder, leading to less membrane rupture than without Ca^2+^. On contrary, if the peptides already aggregate at the membrane before adding calcium ions, the aggregation and therefore the membrane disruption is increased.^12^ Other metal cations such as Cu^2+^, Fe^2+^ or Zn^2+^ are also interacting with Aβ and researchers investigated them more widely than Ca^2+^.^13,14^ However, one property of these ions is to form complexes with Aβ.^15,16^ On contrary, Ca^2+^ significantly block the interaction between the proteins and the lipid membrane. Because there are less studies investigating Ca^2+^ than Cu^2+^, we only focus on calcium ions in the current paper. The main advantage of Supercritical Angle microscopy is the simultaneous measurement of the bulk solution and molecules that are located close to the surface in two different channels due to separation of the signal coming from the surface above the critical angle. It is simpler to analyze surface bound molecules with this optical method. Furthermore, it is non-invasive to biological samples in aqueous solutions, which makes it applicable in different research areas such as biology, physics, surface-chemistry or material sciences.^17,18^ However, only Supercritical Angle Fluorescence (SAF) has a high enough intensity to measure under real biological conditions. The Raman scattering cross-sections is typically ∼10^−29^ to 10^−31^ cm^2^,^19^ which is lower than the fluorescence cross-section. Specifically when we deal with biological samples, the low concentration is a critical factor because of weak sensitivity of Raman spectroscopy. The advantage of conventional Raman spectroscopy over fluorescence is the label-free method, which is suitable to measure the secondary structure of proteins. The Raman scattering suffers low signal-to-noise (SNR) ratio. Therefore, classical assays such as ThioflavinT (ThT), circular dichroism (CD) and transmission electron microscopy (TEM) can be useful for Aβ protein characterization.^20,21^ Note that the characterization of Aβ proteins interacting with cations and nanoparticles using TEM have been reported.^22,23^ However, these methods allow measurements only in one environment, either with lipids or without lipids. Therefore, the direct comparison of proteins far and near of the membrane is not possible. Note that the supercritical angle Raman and supercritical angle fluorescence offers simultaneous measurement of the bulk solution and the surface. With this method, the structural changes near the lipid membrane, which are close to the microscope glass slide, can be observed with reduced photobleaching, improved SNR and axial resolution. One drawback of CD spectroscopy is the lack of high-resolution in complex systems such as proteins interacting with a lipid membrane.^24^ In addition, it is not possible with ThT assays to detect oligomers and protofibrils, since the dye only interacts with fibrils. ThT may also influence the aggregation of Aβ, which falsifies the results of the experiments.^25^

V. Dubois *et al.*^26^ used SAF microscopy to investigate the interaction between Aβ peptides and a solid supported lipid bilayer (SLB). The results showed a higher aggregation rate of Aβ_1–42_ compared to Aβ_1–40_. Furthermore, Aβ_1–42_ is more likely to bind to the SLB as Aβ_1–40_ and have a higher oligomerization. V. Dubois *et al.*^27^ also studied the interaction between Aβ and the SLB with supercritical angle Raman spectroscopy (SAR^28^), a label-free technique, which resolves the secondary structures in proteins and peptides. Their results showed a major number of β-sheets in Aβ_1–40_ whereas Aβ_1–42_ exploits more β-sheets in the bulk solution, while α-helices bind stronger to the surface of the lipid membrane.

In this current work, the interaction between the two Aβ peptides and a SLB is investigated with supercritical angle Raman (SAR) and fluorescence (SAF) spectroscopy and microscopy to study the aggregation at the surface compared with the bulk solution. The SLB is an easy membrane model, which is reproducible and therefore widely used to study the mechanism of the Aβ aggregation on membranes.^26,29,30^ In addition, the influence of calcium ions on the Aβ aggregation is studied. The aggregation of both peptides is compared, since some differences are already known, also with regard to AD: Aβ_1–40_ has a larger abundance, Aβ_1–42_ is more hydrophobic and more neurotoxic. In addition, Aβ_1–42_ undergoes more fibrillation than Aβ_1–40_.^2^ The studies were performed *in vitro* to investigate only the direct influence of calcium ions on the Aβ aggregation and omitting all other interactions within the cells.

## Experimental

### Materials

Aβ_1–40_ (APExBIO, Houston, Texas, United States), Aβ_1–42_ (Sigma-Aldrich, Pennsylvania, United States), ATTO633-COOH (ATTO-TEC, Siegen, Germany), 1,2-dioleoyl-*sn-glycero*-3-phospho-choline (DOPC, Avanti Polar Lipids, Alabaster, Alabama, United States) in chloroform, 1,2-dioleoyl-*sn-glycero*-3-phospho-l-serine (DOPS, Avanti Polar Lipids, Alabaster, Alabama, United States) in chloroform, CaCl_2_·2H_2_O (Sigma-Aldrich CHEMIE, Steinheim Germany), Dulbeccos PBS (Sigma-Aldrich, Buchs, Switzerland), NaCl (Sigma-Aldrich, Buchs, Switzerland), tris(hydroxymethyl)aminomethane (tris, Sigma-Aldrich CHEMIE, Steinheim Germany), Nuclepore Track-Etch membrane (0.1 μm pore size, Whatman, Merck, Darmstadt, Germany), cover glass (MENZEL-Gläser, Braunschweig, Germany), Loctide 3105 (Henkel, Erlinsback, Switzerland), Deconex cip7 (Avan-tor, Dietikon, Switzerland), EtOH (REUSS-CHEMIE AG, Tägerig, Switzerland), Milli-Q-H_2_O (MQ H_2_O, deionisied H_2_O, filtered in Milli-Q system, Merck, Darmstadt, Germany), custom made metal plate (university intern workshop).

### Methods

#### SLB preparation

DOPC and DOPS were used in a mixture of 65 : 35 (DOPC : DOPS). In earlier SLB-peptide studies, this ratio was optimized.^31^ The lipids (solution in chloroform) were vortexed, stirred under nitrogen and completely dried under vacuum (20 mbar) over night. Then, the lipids were mixed with 1 mL membrane buffer (100 mM NaCl, 5 mM CaCl_2_·2H_2_O, 10 mM tris, pH 7.4, filtered, degassed) and vortexed. To get homogenously distributed unilamellar vesicles of the same size, the suspension was extruded 25 times through a millipore membrane with 0.1 μm pore size and subsequent diluted to 0.1 mg mL^−1^ with membrane buffer. To build the measurement plate, a glass coverslip was washed in an ultrasonic bath with Deconex cip7, then EtOH and at the end with Milli-Q H_2_O and glued afterwards to the metal plate, which was washed in the same way. The liposomes were added to the measurement plate, which is connected to a peristaltic pump, for 15 min (200 μL min^−1^) to reach the critical concentration for fusion to the SLB on the 0.16 mm thick cover glass. The remaining vesicles were removed by washing with the membrane buffer at the same flow rate for 45 min and the SLB was stabilized for at least one hour in Dubelcos PBS before the measurements.

#### Peptide preparation

The Aβ peptides Aβ_1–40_ and Aβ_1–42_ were used in their monomeric form as a powder with a purity ≥ 95% (HPLC). The peptides were dissolved in PBS under stirring at 300 rpm, for 30 min at 20 ± 1 °C and diluted to a concentration of 1 mg mL^−1^ and stored at −20 °C until use. This concentration correlates to 230 μM Aβ_1–40_ and 222 μM Aβ_1–42_. Each protein was either incubated in 50% v/v Milli-Q H_2_O or 25 mM CaCl_2_·2H_2_O in Milli-Q H_2_O, resulting in a total concentration factor of 0.5. Therefore, the total concentration of Aβ_1–40_ is 115 μM and of Aβ_1–42_ is 111 μM. The final PBS concentration is 2.4 mg mL^−1^. The same was done with the fluorescent peptides with a subsequent staining with 10 μL of 0.1 mg mL^−1^ ATTO633-COOH in PBS. ATTO633-COOH was used, since absorption spectrum of the dye is well suited for the excitation laser wavelength. It has a high fluorescent yield, a high thermal and photo-stability and it is suitable for single molecule fluorescence spectroscopy such as SAF/UAF. Compared to other dyes, ATTO633-COOH is rather small in size and low molecular weight, which would influence less the interactions of the peptides.

#### Optical setup

Our experimental setup is based on custom-made objective with a parabolic shaped lens, which collects the signal above the critical angle and has been introduced in previous works of the group.^18,27,32^ In short, a diode laser serving at wavelength 633 nm (TOPTICA iBeam Smart; TOPTICA Photonics AG, Germany) was used as an excitation light source. The laser beam was guided into the inverted microscope (Olympus Europa GmbH, Germany) and focused by the oil immersion objective lens with a numerical aperture 1. The supercritical and undercritical signals were separated by a 45-degree mirror in the back-reflection mode. For Raman signal measurements, the SAR and UAR signals were collected by a multimode fiber, and finally detected by the spectrometer equipped with a low-noise CCD camera cooled to −70 °C. Similarly, for fluorescence measurements, the separated signals were detected by two identical single photon avalanche diodes (SPADs).

#### SAR/UAR measurements

The SAR/UAR measurements were performed on the set-up as described above. The photons near to the surface (∼200 nm) are collected in the SAR channel and the photons from the bulk solution up to ∼2.5 μm in the UAR channel. The height of the objective was set in a way, that the focal point is above the glass slide. The laser power was set to 50 mW for the Raman measurements (the laser power after the objective is 16 mW), each three measurements of three different points in the sample were recorded with a 100 μm slit width at the detector for the UAR measurements and a 200 μm slit width for SAR measurements, an acquisition time of 60 s and the signal was accumulated over 5 spectra. The spectra were recorded at 20 ± 1 °C. For data analysis, all the data were smoothen first using the Savitzky–Golay approach with a second order polynomial degree and ten points. The baseline was subtracted from the data acquired for the samples to reduce the background and artefacts from the measurements. The baseline subtraction was performed with the MATLAB (MathWorks, Natick, Massachusetts, United States) program ‘Baseline’.^33^ The spectra were normalized to the maximum and averaged. The measurements were performed in LabSpec5 (HORIBA Jobin Yvon GmbH, Oberursel, Germany). The smoothing, normalization and averaging, as well as the peak analysis (deconvolution into Gaussian peaks and integral calculations) were performed using OriginPro (OriginLab, Northampton, Massachusetts, United States).

#### SAF/UAF measurements

The SAF/UAF measurements were performed on the set-up as described above to allow a simultaneous collection of both fluorescence signals. The area of interest was scanned by a remotely controlled moving frame of the microscope, using a Borlean C++ program, written by the group.^31^ Images with an area of 150 × 150 μm^2^ size were selected. This corresponds to 240 × 240 pixel with a pixel size of 625 nm. The intensity signals of the scans were normalized to the maximum and plotted with MATLAB (MathWorks, Natick, Massachusetts, United States) and the resulting images were analysed using ImageJ (National Institutes of Health, Bethesda, Maryland, United States). The intensities and lengths of the aggregates were measured. The imaging contrast (*C*) of the SAF/UAF images were calculated with eqn (1),^34^ to get the signal to background ratio.1
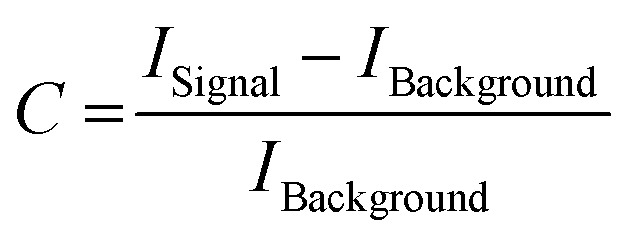


## Results and discussion

**Fig. 1:**
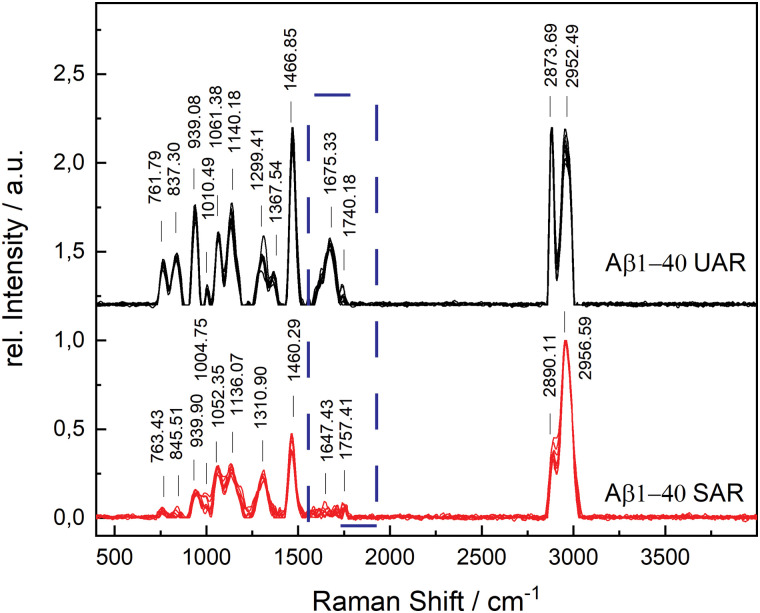
Difference of SAR (red) and UAR (black) spectra of Aβ_1–40_ incubated for 72 h at 20 ± 1 °C in MQ H_2_O and measured on the SLB. All the measured spectra are shown to display the spectral variability and were normalized to the highest peak intensity. Peak assignments in cm^−1^: 762/763 and 837/846: Tyr, 939/940: *ν*(N–Cα–C) and Val/Ile, 1010/1005 and 1061/1052: Phe ring, 1140/1136: C–N, 1299/1311: amide III, 1368/—: *δ*(Cα–H), 1467/1460: *δ*(C–H), 1610/1615: Phe 1675/1647: amide I, 1740/1757: Glu/Asp, 2874/2890 and 2952/2957: *ν*(C–H). The largest difference between UAR and SAR is in the range between 1530 and 1800 cm^−1^ (dark blue rectangle), which is also the most sensitive region in the protein Raman spectrum.

**Fig. 2:**
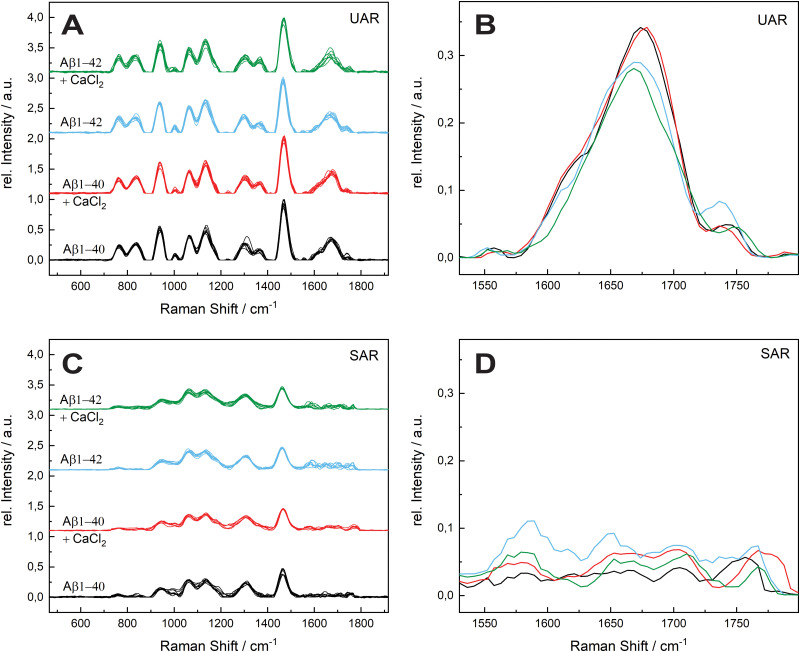
Comparison of the effect of calcium ions on the two forms of Aβ after 72 h. (A) Undercritical Angle Raman (UAR) spectra of 115 μM Aβ_1–40_ incubated in MQ H_2_O for 72 h at 20 ± 1 °C (black), 115 μM Aβ_1–40_ incubated with 25 mM CaCl_2_ for 72 h at 20 ± 1 °C (red), 111 μM Aβ_1–42_ incubated in MQ H_2_O for 72 h at 20 ± 1 °C (blue) and 111 μM Aβ_1–42_ incubated with 25 mM CaCl_2_ for 72 h at 20 ± 1 °C (green). (B) Magnification of the mean value of the spectra from A on range of the amide I band (1530–1800 cm^−1^). The spectra are overlaid for better comparison and the color assignment is the same as in A. (C) Supercritical angle Raman (SAR) spectra of the same samples as in A with the magnification of the amide I band (mean values) (D). All samples were measured on a SLB and normalized to the maximum peak intensity. The spectral variability is higher in the SAR spectra and within the spectra the highest variability is in the amide I region.

The aggregation time was set to 72 h, since A. Itkin *et al.*^35^ observed the first major difference after this time, and it is important to discover the aggregation after a long time. Fig. 1 shows the UAR (black) and SAR (red) spectra of Aβ_1–40_ incubated for 72 h at 20 ± 1 °C in PBS (2.4 mg mL^−1^) and measured on the SLB. The two spectra differ from each other due to the interaction with the lipid membrane in the SAR channel. Most peaks in the SAR channel have a lower intensity, since the SAR method is less sensitive to the vibrational changes in the molecules than the UAR. The Raman shifts of each single peak are also slightly different in the UAR and SAR. The peak assignment to chemical bonds or amino acids/structures are as following: 762/763 and 837/846: Tyr, 939/940: *ν*(N–Cα–C) and Val/Ile, 1010/1005 and 1061/1052: Phe ring, 1140/1136: C–N, 1299/1311: amide III, 1368/—: *δ*(Cα–H), 1467/1460: *δ*(C–H), s1610/1615: Phe, 1674/1647: amide I, 1740/1757: Glu/Asp, 2874/2890 and 2952/2957: *ν*(C–H). All Raman shift values are represented in cm^−1^. The spectral variability is higher in the SAR than in the UAR spectrum due to the lower sensitivity of the SAR channel. Within the spectra, the largest variability is between 1550 and 1700 cm^−1^, in the amide I region (marked as a dark blue rectangle, Fig. 1). This region also differs the most between UAR and SAR. Due to the interaction with the lipid membrane, some peaks are shifted, some are not visible anymore and some new peaks arise. Mostly the peaks of hydrophobic amino acids are changed in the SAR due to interaction with the membrane. The hydrophobic Phe peak is almost not visible in the SAR, but it is visible in the UAR. Since both forms of the Aβ are important on the pathway of AD, Fig. 2 gives a comparison of the Raman spectra of both peptides with and without CaCl_2_. Fig. 2(A) shows the UAR spectra of Aβ_1–40_ (black), Aβ_1–40_ with CaCl_2_ (red), Aβ_1–42_ (blue) and Aβ_1–42_ with CaCl_2_ (green). All samples were measured on a SLB and incubated for 72 h without the SLB, before adding to the SLB for the measurements. Only the most relevant part of the spectra (465–1917 cm^−1^) is shown, since the spectra are normalized to the vibrational stretch of the C–H bond and therefore this band is the same for all spectra. The peak assignment is the same as above with some slight shifts in the wavenumbers. In Fig. 2(B), the spectra from Fig. 2(A) are magnified and overlaid for better comparison of the amide I peaks (1530–1800 cm^−1^).

The UAR spectra of Aβ_1–40_ are similar, no matter if CaCl_2_ is added or not. The Raman shifts of the amide I band are similar in both spectra, meaning the secondary structure remains the same, when it aggregates in the presence of calcium ions for 72 h. The main peak is at 1669.36 ± 2.51 cm^−1^ for Aβ_1–40_ without calcium and 1667.84 ± 3.65 cm^−1^ for Aβ_1–40_ with 25 mM CaCl_2_, meaning the peptides possess mainly β-sheet structures with a small shoulder in the α-helix region at 1621.55 cm^−1^. The intensity of the amide I peak is a bit increased, when aggregating in the presence of calcium ions. This result shows clearly that β-sheets are formed after 72 h of aggregation, even when calcium ions are present. Therefore, calcium does not prevent Aβ_1–40_ from aggregating in the bulk solution, since the aggregation after 72 h is almost the same as without calcium ions. The Raman measurements were also carried out with different concentrations of CaCl_2_ and Aβ. The results are presented in the ESI.†

The amide I band (UAR) of Aβ_1–42_ differs more when aggregated 72 h in the presence of CaCl_2_ compared to Aβ_1–40_. It also has the highest spectral variability of all samples. This band is flatter, but has on average more or less the same central wavenumber of the β-sheet peak (1667.19 ± 4.31 cm^−1^). Some of the single spectra are shifted slightly to lower wavenumbers. A lower wavenumber and a higher relative intensity mean a stronger bond in the beta sheet structure of Aβ_1–42_ in the bulk solution and therefore a stronger aggregation.

Fig. 2(C) shows the SAR spectra of the same samples with the same color code and same range in Raman shift as in Fig. 2(A). The magnification of the amide I region is presented in Fig. 2(D). The spectrum of Aβ_1–40_ (black) has a peak at 1680.42 ± 16.60 cm^−1^, indicating a β-sheet as in the UAR spectrum with a slightly higher shift, meaning a slightly weaker β-sheet at the surface. In addition, there is a peak in the α-helix range at 1652.16 ± 9.90 cm^−1^. When CaCl_2_ is added (red) for incubation, most of the peaks of the whole spectrum have a lower relative intensity and there is a shift of some individual peaks to higher wavenumbers. This result shows less aggregation with weaker bonds of Aβ_1–40_ at the surface of the lipid membrane, when incubated for 72 h in CaCl_2_ containing conditions. The Glu/Asp peak has a stronger shift to higher wavenumbers, compared to the rest of the spectrum, resulting in an ester bond at 1780 cm^−1^. This ester may result through binding to other amino acids or to lipids. One possible reason for this ester bond could be the reaction between the laser-activated lipid head groups and the calcium binding glutamate or aspartate. The amide I band of Aβ_1–42_ (blue) has a higher intensity as of Aβ_1–40_ in both, the β-sheet (1682.96 ± 15.65 cm^−1^) and the α-helix region (1649.70 ± 6.32 cm^−1^). However, the increase in the α-helix is much more intense than the β-sheet increase. This means, Aβ_1–42_ forms mostly stronger α-helices at the surface to get anchored into the lipid membrane, but also some β-sheets. In the bulk, Aβ_1–42_ forms only β-sheets. In contrast, Aβ_1–40_ forms more β-sheets over the whole space. This fact is consistent with the fact, that Aβ_1–42_ is more cytotoxic than Aβ_1–40_.^1,36^ When incubated for 72 h together with CaCl_2_ (green), there is not a large shift difference, but a decrease of intensity in the amide I region (1530–1800 cm^−1^), therefore there is less aggregation at the surface.

**Fig. 3:**
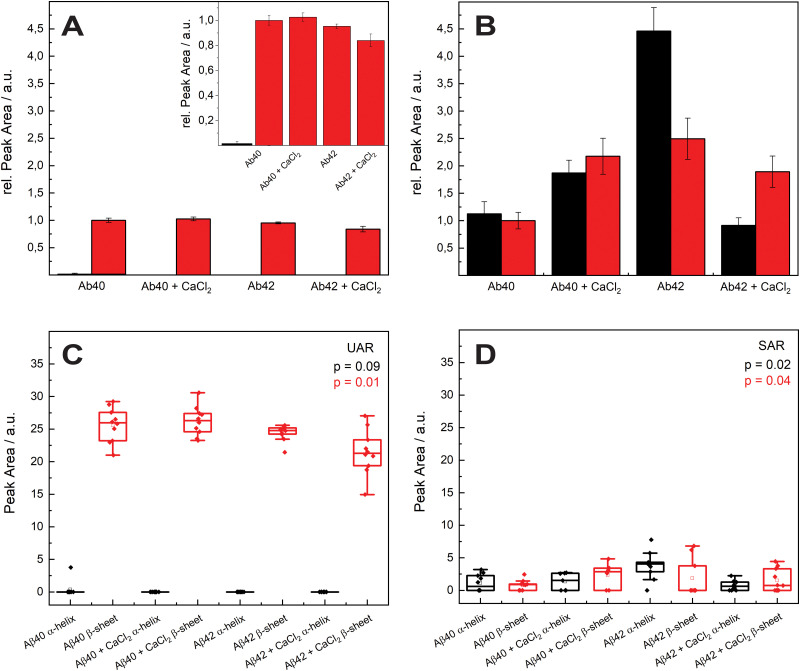
Mean values of the Integrals of the amide I peak (1640–1700 cm^−1^) of all samples. (A) The relative peak area of the amide I region (1640–1700 cm^−1^) in the UAR for the samples of 72 h incubation time. The black and red bars represent the α-helix and β-sheet peaks. The inset is a zoom-in for a better comparison of each bar. (B) The relative peak area of the amide I region (1640–1700 cm^−1^) in the SAR. The error bars are the standard deviations of each sample. (C) and (D) Represents the box charts of the absolute integrals of the amide peaks for the UAR and the SAR respectively. The statistically significance given by the *p*-value, which is lower than 0.05 (exception: UAR α-helix, due to an outlier). The UAR is slightly more significant than SAR, since SAR is more sensitive to the surface and little fluctuations may influence the values more than in the UAR, however the significance is still given by *p* < 0.05. The black and red boxes represents the α-helices β-sheets.

In Fig. 3, the mean values of the integrals of the deconvoluted amide I peaks for the spectra in Fig. 2 are shown to compare the number of α-helix and β-sheet bonds that are present in each sample. They are normalized to the integral of the β-sheet of Aβ_1–40_ without calcium ions for comparison for both UAR and SAR. Fig. 3(A) shows the integrals from the UAR spectra, the inset is a zoom-in for a better comparison of each bar, and similarly, Fig. 3(B) shows the integral from the SAR spectra. When the two conditions of Aβ_1–40_ after 72 h are compared, the peak area with CaCl_2_ increase in the UAR and in the SAR compared to the calcium free aggregation. There is a stronger aggregation, which is also described in the literature.^37^ Together with the higher shift in Fig. 2, Aβ_1–40_ incubated in CaCl_2_ forms more but weaker β-sheets than without CaCl_2_. Because calcium ions block some of the interactions between Glu/Asp and Lys, the β-sheets get weaker, since they are not bond tightly. However, due to more charges, they can be accumulated more easily, reaching higher integrals. As shown by Y. Yang *et al.*,^11^ most of the calcium ions stay near the surface close to the negatively charged lipids, hindering lysine to approach the membrane and therefore hindering aggregation at the surface of the membrane. The integral of Aβ_1–42_ reveals a much stronger aggregation with a lot more of α-helices at the surface than Aβ_1–40_.

These findings are consistent with the Raman shifts of the spectra, since Aβ_1–42_ forms mostly α-helices at the surface which leads to higher aggregation at the SLB due to anchoring of α-helices into the membrane.^38,39^ When Aβ_1–42_ aggregated in the presence of calcium ions, the relative peak intensity of the α-helix drastically decreased. Therefore, Aβ_1–42_ forms more β-sheets at the surface than α-helices when incubated with CaCl_2_. The integral of β-sheets is also a bit lower, when incubated in calcium ions containing conditions. This means that the barrier function of calcium ions works when Aβ_1–42_ approaches the membrane. With Aβ_1–40_ the barrier effect is not working well, probably since the peptide is more hydrophilic than the 42 amino acid variant.

Since Ca^2+^ is blocking the electrostatic attraction, which is the first important step in aggregation at the lipid membrane, the hydrophobic amino acids bury less into the membrane. Therefore, this effect is larger in the more hydrophobic Aβ_1–42_.

**Fig. 4:**
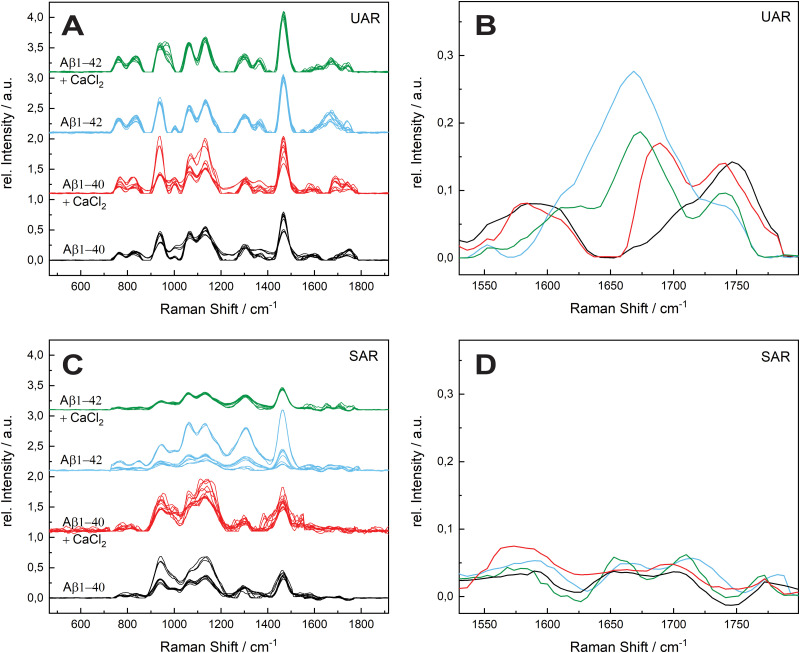
Comparison of the effect of calcium ions on the two forms of Aβ after 96 h. (A) Undercritical Angle Raman (UAR) spectra of 115 μM Aβ_1–40_ incubated in MQ H_2_O for 96 h at 20 ± 1 °C (black), 115 μM Aβ_1–40_ incubated with 25 Mm CaCl_2_ for 72 h at 20 ± 1°C (red), 111 μM Aβ_1–42_ incubated in MQ H_2_O for 96 h at 20 ± 1 °C (blue) and 111 μM Aβ_1–42_ incubated with 25 Mm CaCl_2_ for 96 h at 20 ± 1 °C (green). (B) Magnification of the mean value of the spectra from A on range of the amide I band (1530–1800 cm^−1^). The spectra are overlaid for better comparison and the color assignment is the same as in A. (C) Supercritical angle Raman (SAR) spectra of the same samples as in A with the magnification of the amide I band (mean values) (D). All samples were measured on a SLB and normalized to the maximum. The spectral variability is higher as in the aggregation after 72 h.

The aggregation process was even prolonged to 96 h incubation time (see Fig. 4). The spectral variability is in general higher after 96 h. A possible reason is a different aggregation time of each different protein in the samples. After 72 h most of the proteins are still in the aggregation process, therefore all the spectra are similar in the UAR. In the SAR channel, the case is a bit different, since the aggregation process takes place without SLB. The aggregated samples were added after the aggregation to the lipid membrane. Each protein can have different interactions with the lipids, no matter, if the aggregation time is 72 or 96 h.

The amide I region is the most important, therefore we concentrate on this region between 1530 and 1800 cm^−1^. The UAR spectra of the different samples after 96 h differs more than after 72 h. Furthermore, the relative intensity of the samples decreases after 96 h. For Aβ_1–40_, there is only a shoulder of the Glu/Asp peak around 1699 cm^−1^, which is still in the β-sheet region. One reason for loosing this peak is the accumulation of peptides. When incubated with CaCl_2_ for 96 h, the Aβ_1–40_ have a β-sheet peak that is shifted to higher wavelengths (1690.33 cm^−1^). This means a weaker bond due to the calcium ions, which hinders an accumulation and strong β-sheet formation. The spectra of Aβ_1–42_ incubated for 96 h with or without CaCl_2_ are similar to Aβ_1–42_ incubated for 72 h. The center of the main peak for both Aβ_1–42_ samples is at 1671 cm^−1^. Since Aβ_1–42_ forms fibrils in an early stage, the aggregation is already done after 72 h and there is not a large change after 96 h. In all spectra, the peak of the Glu/Asp interaction has a higher intensity after 96 h. The negatively charged amino acids get shielded with the positive calcium ions and therefore they interact less with other groups. Therefore, they are more distinct in the Raman spectra.

In the SAR spectra, the relative intensities of the amide I peak are similar after 96 h than after 72 h, even the relative intensities of the other peaks differs in some spectra. The center of the amide I peak for the Aβ_1–40_ without CaCl_2_ is at 1661 cm^−1^, a lower wavelength than after 72 h. This makes sense, since the aggregation after a longer time would be stronger. Incubated with CaCl_2_, the peak is shifted into the other direction towards weaker bonds. The interaction to the lipid membrane gets weaker due to the calcium ions on the lipid membrane. The spectra of Aβ_1–42_ with and without calcium ions are similar as Aβ_1–40_ without CaCl_2_, however the peak intensities are higher with the 42 amino acid variant.

The molecules that are already aggregated formed stronger bonds. The conclusion which is drawn from these results is a slower aggregation of Aβ_1–42_ in presence of calcium ions compared to Aβ_1–40_. This finding is consistent with the results from A. Itkin *et al.*^35^ and R. Lal *et al.*^40^ that Aβ_1–40_ forms first small oligomers, which aggregates further, while Aβ_1–42_ forms long fibrils, which need more time to form. In general, there is less aggregation at the surface when the Aβ was incubated for long time in the presence of calcium ions. This fact supports the theory of Y. Yang *et al.*^11^ that the calcium ions form a protecting layer over the membrane to prevent the peptide approaching towards the membrane, since the positively charged lysine amino acids are no longer attracted by the lipid molecules.

**Fig. 5:**
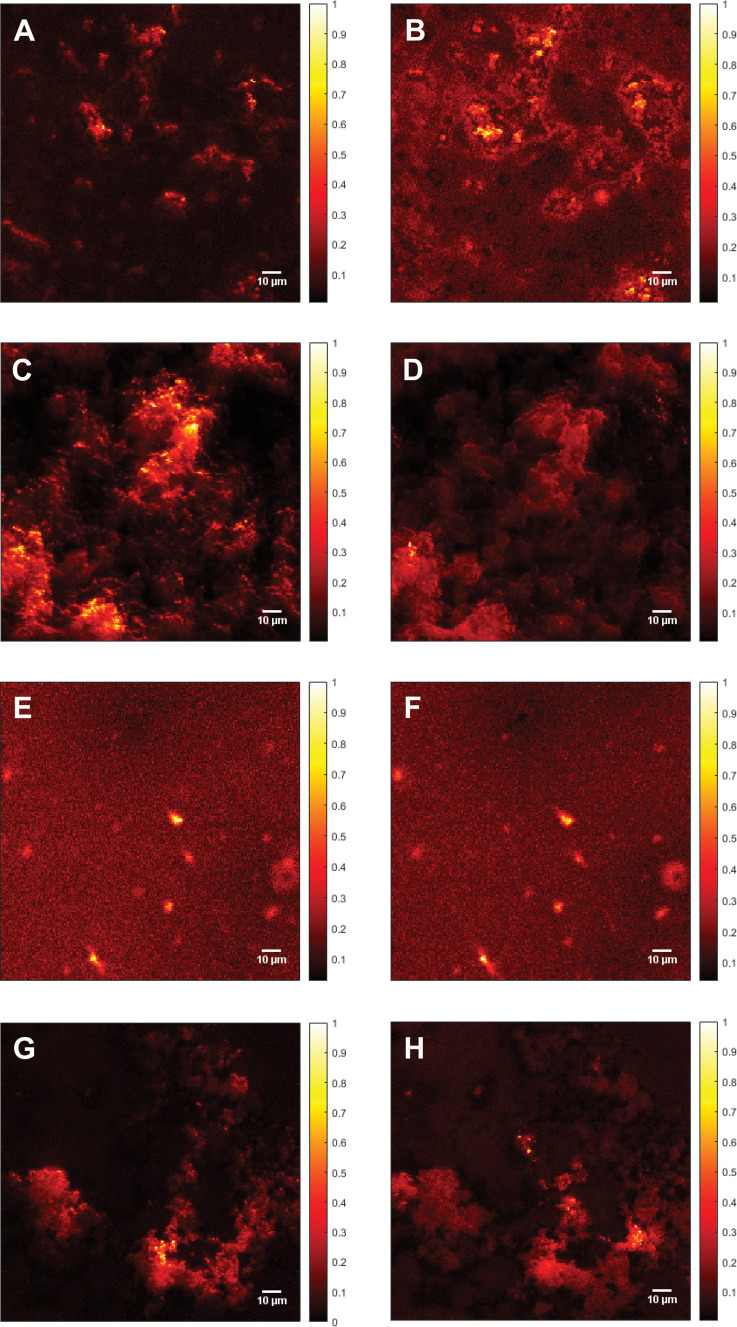
Fluorescence images of Aβ. (A) Supercritical angle fluorescence (SAF) image of 115 μM Aβ_1–40_ incubated for 72 h at 20 ± 1 °C and stained with ATTO633-COOH. Image contrast (calculated according to eqn (1)), *C* = 12.6. (B) Undercritical angle fluorescence (UAF) image of 115 μM Aβ_1–40_ incubated for 72 h at 20 ± 1 °C and stained with ATTO633-COOH, *C* = 7.87. (C) SAF image of 115 μM Aβ_1–40_ incubated with 25 mM CaCl_2_ for 72 h at 20 ± 1 °C and stained with ATTO633-COOH, *C* = 19.3. (D) UAF image of 115 μM Aβ_1–40_ incubated with 25 mM CaCl_2_ for 72 h at 20 ± 1 °C and stained with ATTO633-COOH, *C* = 12.0. (E) SAF image of 111 μM Aβ_1–42_ incubated for 72 h at 20 ± 1 °C and stained with ATTO633-COOH, *C* = 7.27. (F) UAF image of 111 μM Aβ_1–42_ incubated for 72 h at 20 ± 1 °C and stained with ATTO633-COOH, *C* = 11.5. (G) SAF image of 111 μM Aβ_1–42_ incubated with 25 mM CaCl_2_ for 72 h at 20 ± 1°C and stained with ATTO633-COOH, *C* = 17.7. (H) UAF image of 111 μM Aβ_1–42_ incubated with 25 mM CaCl_2_ for 72 h at 20 ± 1 °C and stained with ATTO633-COOH, *C* = 7.90. In the images, the oligomers seem to be around 10 times larger than the actual size due to the diffraction limit. All Samples were measured on a SLB and normalized to the highest fluorescent intensity. The color bars on the right of each image shows the relative fluorescence intensity.

In addition to the Raman spectra, fluorescence images were recorded to further investigate the aggregation process and to give insights into the morphology of the aggregates. Fig. 5(A) shows the SAF image of 115 μM Aβ_1–40_ incubated for 72 h in Milli-Q H_2_O at 20 ± 1 °C and stained with ATTO633-COOH. The average length of all measured aggregates of this sample (on all recorded images) is 6.94 ± 6.53 μm. The size in the images is around 10-fold increased due to the diffraction limit. The 10-fold increase is determined through the 100 nm extruded liposomes, which have a size of around 1 μm in the UAF/SAF. The standard deviation is quite large, since the aggregates differ a lot in size and in shape. However, most of the aggregates have a length between 4 and 7 μm, which is congruent with the mean value of 6.94 μm. The number of aggregates in all images of this sample is *n* = 60. The image contrast was calculated using eqn (1), *C* = 12.6, using the aggregate with the highest intensity for *I*_signal_.

The UAF image (Fig. 5(B)) of 115 μM Aβ_1–40_ shows similar aggregates as the SAF (Fig. 5(A)), but a higher fluorescent background. The average length is 6.55 ± 2.45 μm (*n* = 145), resulting in similar aggregates in the SAR and the UAR. However, the UAR consists of around 2.5-fold the aggregates of the SAR. The UAR image have a lower contrast of *C* = 7.87, which means less aggregation in the bulk. The background fluorescence is higher in the bulk, since the UAF signal is stronger and more fluorescence in general can be detected in the UAF channel than in the SAF channel. Therefore, it is important to do the background correction and use the imaging contrast rather than the intensity itself. The aggregation at the surface is supported by negatively charged lipids, therefore the measured aggregation in the SAF channel is higher.

The fluorescence images of 115 μM Aβ_1–40_ incubated for 72 h in 25 mM CaCl_2_ and stained with ATTO633-COOH differ from them without CaCl_2_ (see Fig. 5(C) and (D)). In the SAF image (Fig. 5(C)), there are more abundant (*n* = 340) and smaller oligomers, which are close together with a size of 3.33 ± 1.36 μm and an intensity of 60.0 Cnts. The image contrast was calculated to *C* = 19.3. This image contrast is a bit higher as without calcium ions, consisting with a larger integral in Fig. 3(B).

As shown in the UAF image (Fig. 5(D)) the aggregates have an average length of 3.36 ± 1.72 μm and an intensity of up to 244 Cnts with an image contrast *C* = 12.0. The number of aggregates is lower as in the SAF channel (*n* = 190). Comparing the aggregates of Aβ_1–40_ incubated in 25 mM CaCl_2_ after 72 h of aggregation, the Aβ dimers and small oligomers accumulates to larger clusters (see Fig. 5(C) and (D)). That seems to be loosely bound and spread over almost the whole surface with probably weak binding to the surface.

The calculated contrast values of the four different images of Aβ_1–40_ shows the highest fluorescence intensity for the aggregates of Aβ_1–40_ with CaCl_2_ at the surface, followed by only Aβ_1–40_ at the surface. The lowest contrast value was calculated for Aβ_1–40_ incubated in H_2_O in the bulk solution. This means the aggregation with calcium ions is in general higher than without, but the aggregates cannot really stick to the surface, since the bonds are weakened due to the charges of the calcium ions. To have a comparison between the SAF and the UAF signal, the ratio *r* = SAF/UAF was calculated. *r*_Aβ_1–40__ = 1.6 and *r*_Aβ_1–40_+CaCl_2__ = 1.6, the aggregation is similar with or without CaCl_2_.

Fig. 5(E) shows the SAF image of Aβ_1–42_ incubated for 72 h in MQ H_2_O. The imaging contrast is 7.27 and the mean value of the length is 6.02 ± 3.21 μm, *n* = 75. In the UAF channel (Fig. 5(F)); the same aggregates are visible as in the SAF image, since the signal from the surface is also detected in the UAF up to a small proportion. The aggregates are similar in the UAR as in the SAR (*C* = 11.5, *l* = 6.93 ± 3.58 μm, *n* = 75). When aggregated in the presence of CaCl_2_ for 72 h (Fig. 5(G) and (H)), the aggregates are slightly larger (7.25 ± 4.22 μm, SAF and 9.61 ± 4.96 μm for UAF) with a much higher amount of these aggregates (*n* = 125 for SAF and *n* = 255 for UAF), but with a lower intensity in general (*C*_SAF_ = 17.7 and *C*_UAF_ = 7.90 for UAF). This result shows that the peptides cannot be anchored strongly into the membrane, when CaCl_2_ is present, due to this positively charged barrier above the membrane, therefore the peptides are spread more widely over the whole lipid bilayer, resulting in more lager aggregates with lower intensity.

A possible model for the aggregation on a molecular level is proposed. These models are based on the results of this work and the mechanism suggested by L. Yu *et al.*,^41^ R. Lal *et al.*^40^ and Y. Yang *et al.*^11^ The first step is the attraction of the lysine group, following by a subsequent insert of hydrophobic residues such as phenylalanine inside the inner part of the membrane. Due to the hydrophobic interactions, the peptide is anchored into the membrane. The calcium ions prevent this insertion into the membrane.

## Conclusions

**Fig. 6:**
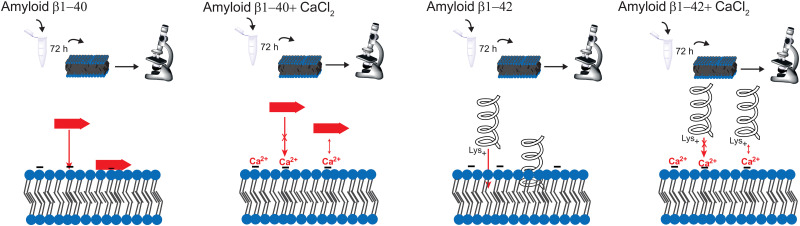
Schematic representation of the aggregation of Aβ. From left to right: Aβ_1–40_ forms mostly β-sheets on the surface. However, the calcium ions block the protein insertion into the membrane. Aβ_1–42_ forms more α-helices on the membrane, which inserts deeper into the membrane than β-sheets. This leads to a stronger effect of calcium ions blocking the insertion of Aβ_1–42_ into the membrane.

In summary, this paper demonstrates the optical spectroscopy and microscopy technique for a better understanding of the aggregation mechanism of Aβ with calcium ions. Even when the spectral variability is higher in the SAR, the method is still reliable and a good tool to investigate proteins at the lipid surface. However, the aggregation process with calcium is much more complicated, than described here, *e.g.* ion channels are formed through the membrane due to Aβ aggregation. The purpose of this paper is to simply present a comparison of the effect of calcium ions between the bulk solution and the surface. Our results show the time dependent Aβ ggregation in presence and absence of calcium ions. Calcium ions enhance the aggregation of Aβ_1–42_; however, it hinders the insertion into the membrane. The reason is the prevention of α-helix formation due to electrostatic hindrance. In addition, there is no strong aggregation at the surface, the peptides are spread over the whole membrane, forming large clusters. The effect of calcium ions is thus larger for Aβ_1–42_, since Aβ_1–40_ forms mostly β-sheets at the surface. A schematic representation of the effect of CaCl_2_ on amyloid β in different aggregation times is depicted in Fig. 6. Hence, the regulation of Ca^2+^ homeostasis is crucial in the beginning of the AD progress.

Of course, *in vitro* aggregation cannot be directly translated in *in vivo* situation, since for the interaction with *e.g.* organelles cells are needed. Admittedly, the proposed models can be used to study the aggregation mechanism. Further studies will provide more information about the *in vivo* investigations with different Ca^2+^ concentrations. A detection system to monitor peptide aggregation inside AD patient's brains would also be helpful. For future work, the positive effect on Aβ aggregation, namely the barrier function could be enhanced by finding a way to prevent aggregates inserting into the membrane before they interact with calcium ions.

## Author contributions

The manuscript was written through contributions of all authors. All authors have given approval to the final version of the manuscript.

## Data availability

All the raw data (Raman spectra and Fluorescence images) used in this article can be accessed from OSF repository at osf.io/3ku9n.

## Conflicts of interest

There are no conflicts to declare.

## Supplementary Material

CP-026-D4CP00996G-s001
