# Supplementary material for: Unveiling the effect of CaCl2 on amyloid β aggregation via supercritical angle Raman and fluorescence spectroscopy and microscopy
Source: Phys Chem Chem Phys. 2024 Oct 1;26(41):26266–76. doi: 10.1039/d4cp00996g (PMC11456997; doi:10.1039/d4cp00996g)
Supplement: CP-026-D4CP00996G-s001 [file CP-026-D4CP00996G-s001.pdf]

Supplementary Information (SI) for Physical Chemistry Chemical Physics.
This journal is © the Owner Societies 2024

Electronic Supplementary Information:

Unveiling the effect of CaCl2 on Amyloid β aggregation via Super-critical Angle Raman and Fluorescence spectroscopy and microscopy

Nathalia Simea Münch,[a] *Subir Das*[a] *and Stefan Seeger*[*a]

[a]*Department of Chemistry, University of Zürich, Winterthurerstrasse 190, 8057 Zürich, Switzerland*

[*]*Email: sseeger@chem.uzh.ch*

Figure S1 shows the comparison of the averaged Raman spectra between the incubation with different CaCl2 concentrations. Aβ incubated with a high calcium concentration (2.5 M) shows a complete different spectrum in both peptides as well in UAR and SAR. This is due to the overloading of the detection volume. The total wavenumbers of all measurements of the Raman shift increase slightly for the β-sheets of both peptides in the UAR with higher salt concentration, resulting in weaker bonds. In the intensities, there is no trend in the UAR, since a lot ions are in the solution. In the SAR channel, the spectra at low calcium concentration (< 25 mM) have similar shape and Raman shifts; however, there is a visible change when increasing the salt concentration. This change at 25 mM let assume, that the blocking effect of calcium ions at the surface appears only at high concentrations.

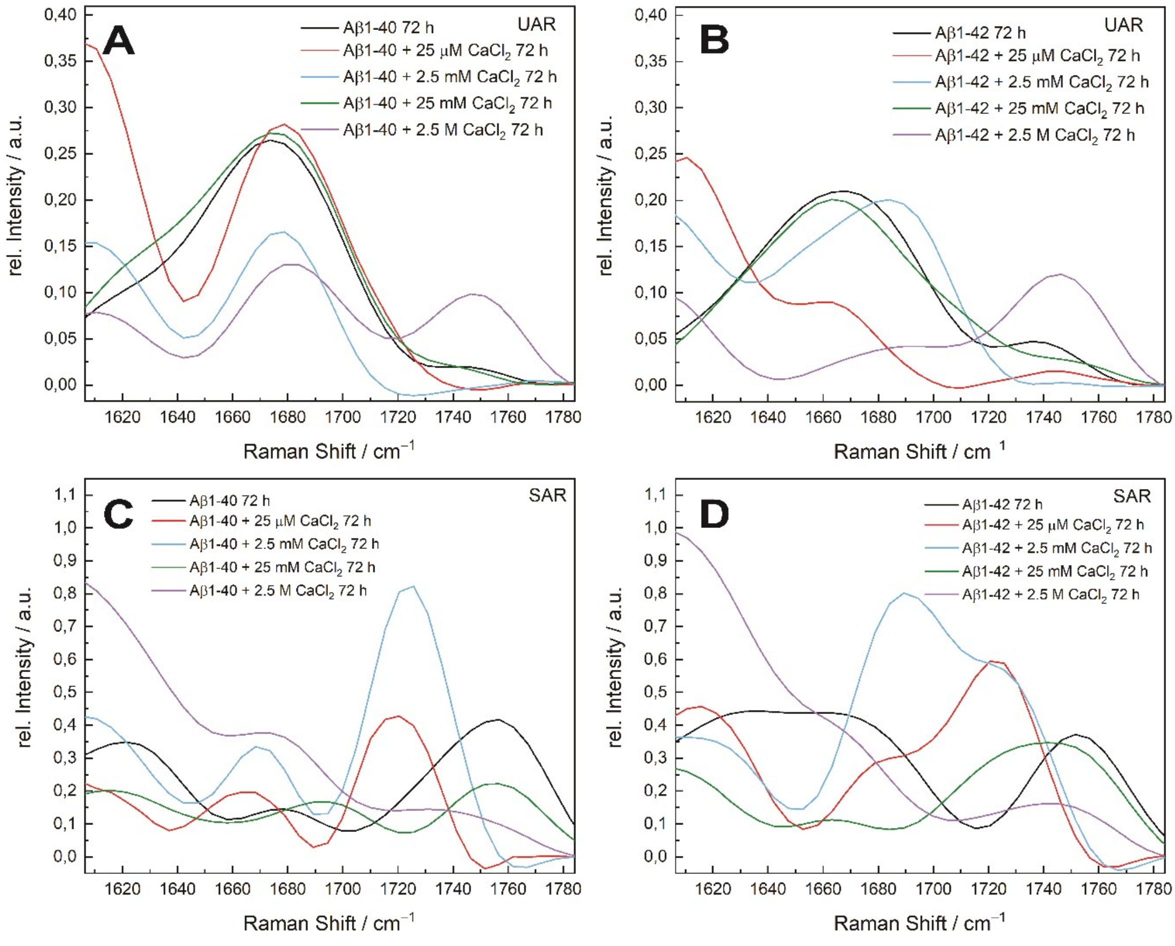

Figure S1*: SAR and UAR measurements of Amyloid β incubating in different CaCl2 concentrations:* ***A****: UAR spectra of Aβ1-40 incubated for 72 h in Milli-Q H2O (black), 25 µM CaCl2 (red), 2.5 mM CaCl2 (blue), 25 mM CaCl2 (green) and 2.5 M CaCl2 (viollet).* ***B****: UAR spectra of Aβ1-42 incubated for 72 h in Milli-Q H2O (black), 25 µM CaCl2 (red), 2.5 mM CaCl2 (blue), 25 mM CaCl2 (green) and 2.5 M CaCl2 (viollet).* ***C****: SAR spectra of Aβ1-40 incubated for 72 h in Milli-Q H2O (black), 25 µM*

CaCl2 (red), 2.5 mм CaCl2 (blue), 25 mм CaCl2 (green) and 2.5 м CaCl2 (viollet). ***D****: SAR spectra of Aβ1-42 incubated for 72 h in Milli-Q H2O (black), 25 μм CaCl2 (red), 2.5 mм CaCl2 (blue), 25 mм CaCl2 (green) and 2.5 м CaCl2 (viollet). The spectra are the mean values of all measured spectra of a given sample.*

Figure S2 shows the validation with 230 μм Aβ1-40 and 222 μм Aβ1-42, which is the double concentration of the conducted experimental concentration. With the higher concentration, there is a higher aggregation; however the trend is the same as in the lower peptide concentrations, which validated the results and making supercritical angle techniques a powerful tool.

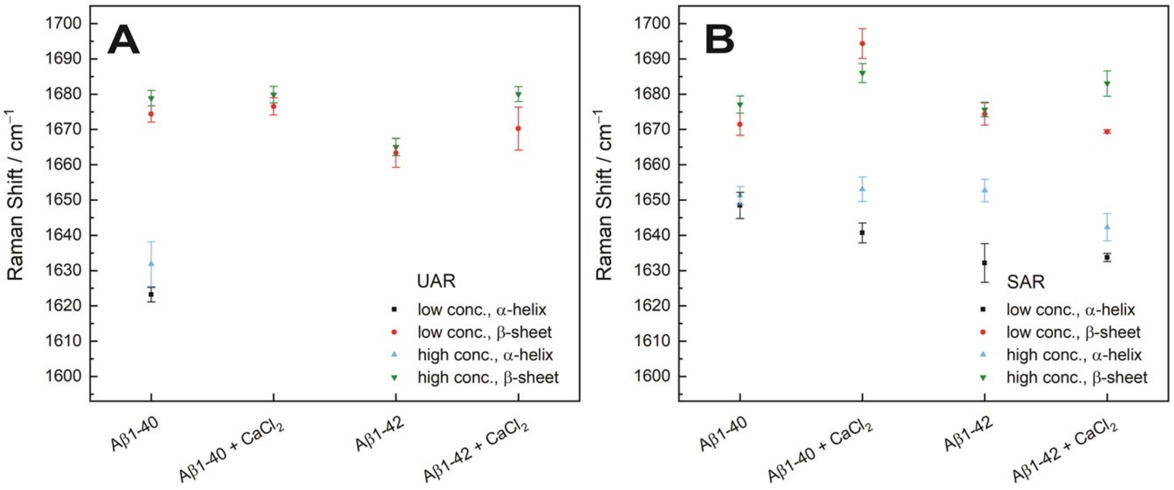

Figure S2: Validation of the results, using the double peptide concentration. ***A****: Mean values of the Raman shifts of the Amid I peaks from the UAR. The black squares are the α-helix peaks of low protein concentration, α-helix, the red dots the low protein concentration β-sheets and the blue and green triangles the α-helix and β-sheets at high peptide concentraion respectively.* ***B****: Mean values of the Raman shifts of the Amid I peaks in the SAR channel.*
